# Targeting TGF-β signal transduction for fibrosis and cancer therapy

**DOI:** 10.1186/s12943-022-01569-x

**Published:** 2022-04-23

**Authors:** Dandan Peng, Minyang Fu, Manni Wang, Yuquan Wei, Xiawei Wei

**Affiliations:** Laboratory of Aging Research and Cancer Drug Target, State Key Laboratory of Biotherapy, National Clinical Research Center for Geriatrics, West China Hospital, Sichuan University, No. 17, Block 3, Southern Renmin Road, Chengdu, 610041, PR Sichuan China

**Keywords:** TGF-β, TGF-β signaling pathways, Targeted therapies

## Abstract

Transforming growth factor β (TGF-β) has long been identified with its intensive involvement in early embryonic development and organogenesis, immune supervision, tissue repair, and adult homeostasis. The role of TGF-β in fibrosis and cancer is complex and sometimes even contradictory, exhibiting either inhibitory or promoting effects depending on the stage of the disease. Under pathological conditions, overexpressed TGF-β causes epithelial-mesenchymal transition (EMT), extracellular matrix (ECM) deposition, cancer-associated fibroblast (CAF) formation, which leads to fibrotic disease, and cancer. Given the critical role of TGF-β and its downstream molecules in the progression of fibrosis and cancers, therapeutics targeting TGF-β signaling appears to be a promising strategy. However, due to potential systemic cytotoxicity, the development of TGF-β therapeutics has lagged. In this review, we summarized the biological process of TGF-β, with its dual role in fibrosis and tumorigenesis, and the clinical application of TGF-β-targeting therapies.

## Background

Transforming growth factor β (TGF-β) is a prototype of the TGF-β family, which is composed of TGF-β, Activin, Nodal, bone morphogenetic proteins (BMPs), growth and differentiation factors (GDFs), and other factors [1, 2]. As a multifunctional polypeptide cytokine, TGF-β plays a critical role in early embryonic development and adult homeostasis [3]. Three subtypes of TGF-β (TGF-βI-III) are only expressed in mammals with unique multifunctional growth factors. In the following paragraphs, TGF-β refers to TGF-βI if not otherwise specified. TGF-β is mainly secreted and stored in the extracellular matrix (ECM) as a latent complex [4], while only activated TGF-β binds to the TGF-β receptor (TβR) complex to lead to its biological functions. Therefore, TGF-β activation is critical for its operation.

In recent years, scientists found that overexpressed TGF-β causes a plethora of metabolic disorders and dysfunction, and promotes epithelial-mesenchymal transition (EMT) and excessive deposition of ECM [5, 6], which causes immune dysfunction, fibrosis, and cancers [7]. Because of the vital function of TGF-β in human fibrosis and cancers, anti-TGF-β approaches have been introduced to treat these diseases [8]. In recent years, many clinical trials have verified the therapeutic effect of TGF-β-targeted drugs on a variety of tumor and fibrotic diseases. By combining TGF-β-targeting drugs (anti-TGF-β antibody, TβR inhibitor, and recombinant proteins) with other antigens (programmed cell death one ligand 1 (PD-L1), M7824, SHR-1701, JS201, TST005, and COX-2 (STP705)) is the most popular treatment strategy currently. This review focuses on the biological process of TGF-β, its dual role in fibrosis and tumorigenesis, and the clinical application of TGF-β-targeting therapeutics.

### The procession of TGF-β

Pro-TGF-β is synthesized as a latent complex in the ECM and is associated with a signal peptide in the large N-terminal portion called the latency-associated peptide (LAP) and a mature cytokine in the C-terminal fragment [9–11]. The large latent complex (LLC) comprises LAP, TGF-β, latent TGF-β binding proteins (LTBP) 1/3, and LTBP4. Latent TGF-β is activated by proteins and enzymes (thrombospondin 1 (TSP-1), glycoprotein A repetitions predominant protein (GARP), integrins, and other TGF-β-binding proteins) and transformed into disulfide-linked dimers and homodimeric ligands. The activated TGF-β interacts with the TβR complex or other cytokines to regulate biological responses through drosophila mothers against decapentaplegic (SMAD) and/or non-SMAD pathways [12].

### TGF-β secretion

**Fig. 1:**
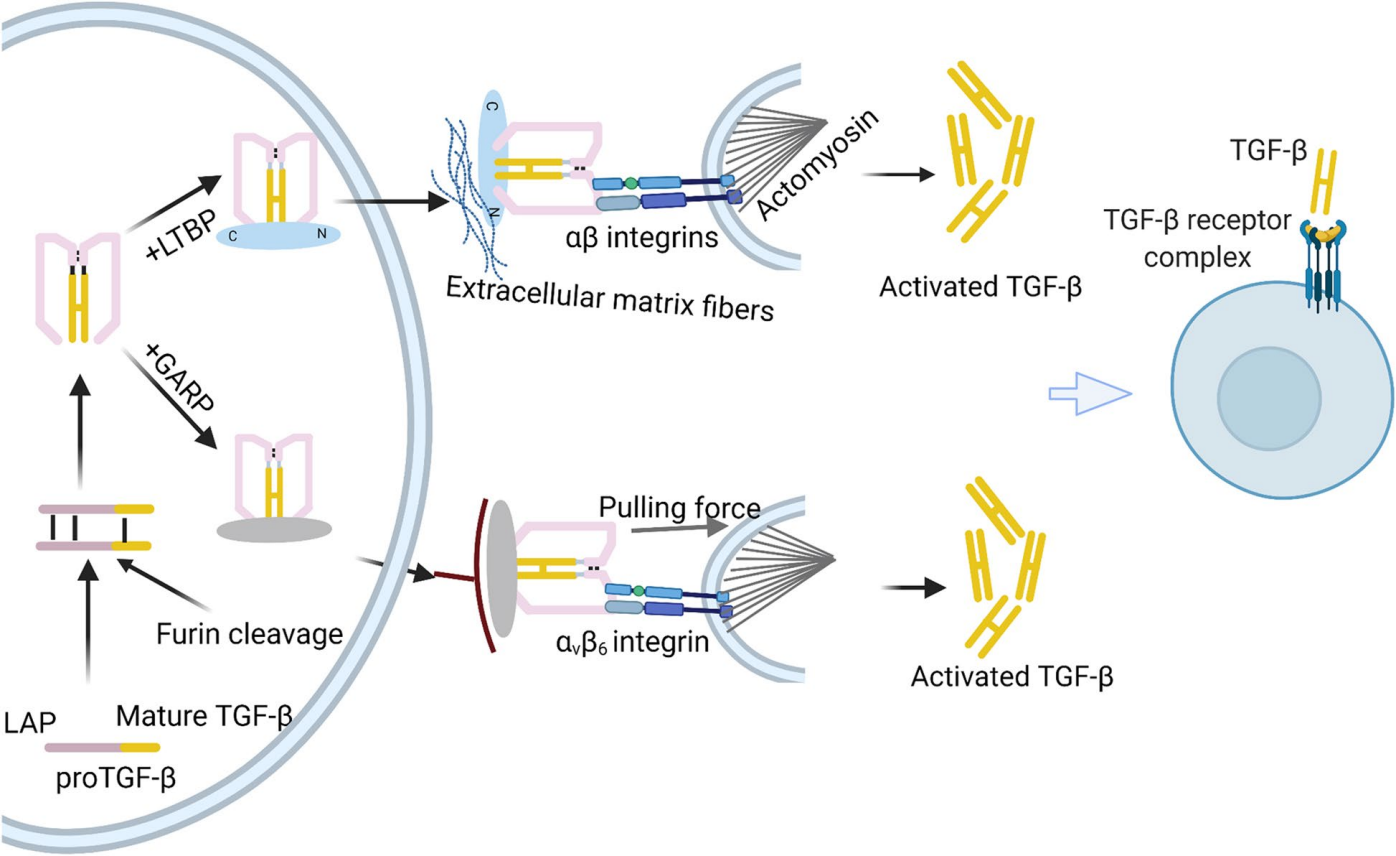
A schematic representation of TGF-β activation The pro-TGF-β synthesized in the rough endoplasmic reticulum becomes latent TGF-β when cleaved by the convertase furin in the Golgi complex. Then the LAP dimer binds to mature TGF-β noncovalently to form a small latent complex (SLC). Then, SLC generally binds to LTBP, forming LLC, while binds to GARP in Treg cells. SLC is anchored to ECM proteins, including fibronectin and fibrillin, via LTBP. Both LTBP and GARP play a direct role in anchoring TGF-β for traction-driven activation by integrins. With the help of αβ integrins and mechanical force, latent TGF-β becomes active and connects to the TβR complex to regulate transcription

LAP binds to LTBPs covalently via two disulfide bonds with two cysteine residues [10]. LTBPs are the promoter of the folding of TGF-β precursor protein. In addition, LTBPs are crucial to latent TGF-β location and activation [13, 14]. LAP, in turn, is cleaved by furin (an indispensable proprotein convertase) from the mature TGF-β precursor in the trans-Golgi network (Fig. 1) [12], in which LTBPs are considered as the primary activator [13]. Although LAP is cleaved from the C-terminal portion, it remains associated with the mature cytokine TGF-β noncovalently [2].

### Location and activation of TGF-β

In general, LLC is secreted to the ECM and is located there via the unique biological properties of LTBP, which interacts with extracellular matrix fibers to keep TGF-β in an inactive form (Fig. 1) [13, 15]. A recent study showed that the crystals of pro-TGF-βI are a ring-shaped complex. When LAP-surrounded TGF-β monomers are freed under cytoskeletal force, the active cytokine interacts with TβR to regulate cellular responses (Fig. 1). This force-dependent activation requires the unfastening of a pro-domain named the ‘straitjacket’ element [16].

### Enzymatic activation

In addition to the nonproteolytic mechanism for the activation of latent TGF-β, proteases are also involved in TGF-β activation. In addition, these proteases are divided into containing glycosidases (N-glycanase and neuraminidase) and serine proteases (plasmin, cathepsin D, and matrix metalloproteases) [10, 17, 18]. Summarily, in the cytoplasmic matrix, TGF-β can be activated by several proteases, integrins, and other TGF-β-binding proteins in different cell types, tissues, and disease milieus [19]. Notably, the proteolytic cleavage sites of latent TGF-β implicate the mechanism of how proteases cleave the LAP latency lasso. For instance, plasma kallikrein (PLK) cleaves residues between R58 and L59 of latent TGF-β [20]. Studies on the activation process of TGF-β at the molecular level helps to targeting-TGF-β therapy.

### Regulation by fibrillin

LTBPs are structurally related to and physically bound to another family of proteins called fibrillins. Fibrillin 1 can function as an inhibitor of TGF-β signaling, but whether it works more directly in controlling the fibrillin-LTBP interaction or stability suppress latent LLC proteolytic activation still needs to be explored [21, 22]. As integral components of microfibrils, fibrillins play different roles in microfibril biology [23]. The microfibrils cover the elastin core of elastin-containing fibers and promote the temporal and spatial regulation of TGF-β activation [24]. Scientists previously suggested that fibrillin-1 can be presented to the surface-exposed loop when binding to the arginine-glycine-aspartic acid (RGD) integrin-binding motif [25–27]. While the remaining fibrillins showed little inhibitory effect on TGF-β activation. A number of studies indicated that fibrillin 2 expression is mainly restricted to developing fetal tissues, while fibrillin-1 expression endures throughout adult life [28]. Moreover, fibrillin-1, together with associated molecules, masks fibrillin-2 epitopes to block its bioactivity. Therefore, fibrillin 1 shows stronger anti-TGF-β activity. Notably, a recent study showed that when local fibrillin-1 was downregulated, fibrillin 2 molecules were exposed in the tumor endothelium with a lower capacity to block TGF-β [29]. Moreover, Heena Kumra et al. suggested that fibrillin-4 might regulate LTBP-4 matrix assembly to impact TGF-β signaling [30].

### Regulation of TGF-β activation by GARP

Recent evidence demonstrated that regulatory T cells (Tregs) could promote latent TGF-β presented by GARP to integrin αVβ8 integrin (Fig. 1) [31]. Unlike LTBPs are abundantly presented in the ECM, GARP is retained only on the surface of Foxp3-expressing Tregs [32]. It is generally accepted that αVβ8 integrin is involved in GARP/TGF-β complex activation, but the exact mechanism is controversial. Some scientists indicated that cytoskeletal force was unnecessary for αVβ8-mediated TGF-β activation. Others believed that the regulation of TGF-β activation by GARP required the release and diffusion of mature TGF-β [33]. In addition, they discovered that mature TGF-β signals were involved in latent TGF-β, which indicated that αVβ8-mediated TGF-β activation may form a large multi-component cell–cell protein complex to induce the SMAD-dependent pathway [34, 35]. Regardless of the mechanism of GARP-induced TGF-β activation, targeting GARP is one of the approaches to avoid TGF-β activation, targeting GARP is one of the approaches to avoid TGF-β activation. Notably, a study showed that monoclonal antibodies against GARP in GARP/TGF-βI complexes could not recognize amino acids GARP137-139 within GARP/TGF-βI complexes could not inhibit Treg-associated TGF-β activation [36].

### Activation of TGF-β by integrins

Integrin family members are implicated in the recognition and activation of TGF-β [37–44]. In addition, integrin-mediated TGF-β activation is essential in the immune system (integrins αvβ6 and αvβ8), tumorigenesis, and fibroblasts. Both Integrins αvβ6 and αvβ8 regulate TGF-β signaling by binding to a linear tripeptide RGD depending on actin cytoskeleton-generated tensile force [45]. In addition to integrins αvβ6 and αvβ8, integrins α8β1, α5β1, and αIIβ3 can also recognize the RGD site in the LAP region of TGF-β. This RGD recognition mechanism regulates the growth and differentiation factors of the TGF-β family to maintain morphogenesis and homeostasis [46].

However, the presence of integrin alone is insufficient for TGF-β activation. Considerable studies have suggested that actin-myosin contraction and mechanical deformation are of great importance for TGF-β activation. In addition, scientists widely believed that the contraction of the actin cytoskeleton previously generated integrin-mediated TGF-β activation by physical force. Furthermore, a study by Melody G. Campbell recently indicated that integrin αvβ6, along with its entire ectodomain, activates GARP to locate latent TGF-β without the release and diffusion of mature TGF-β [47]. In general, identifying a complete regulatory pathway would facilitate the development of more effective therapeutic strategies.

### TGF-β signaling pathways

**Fig. 2:**
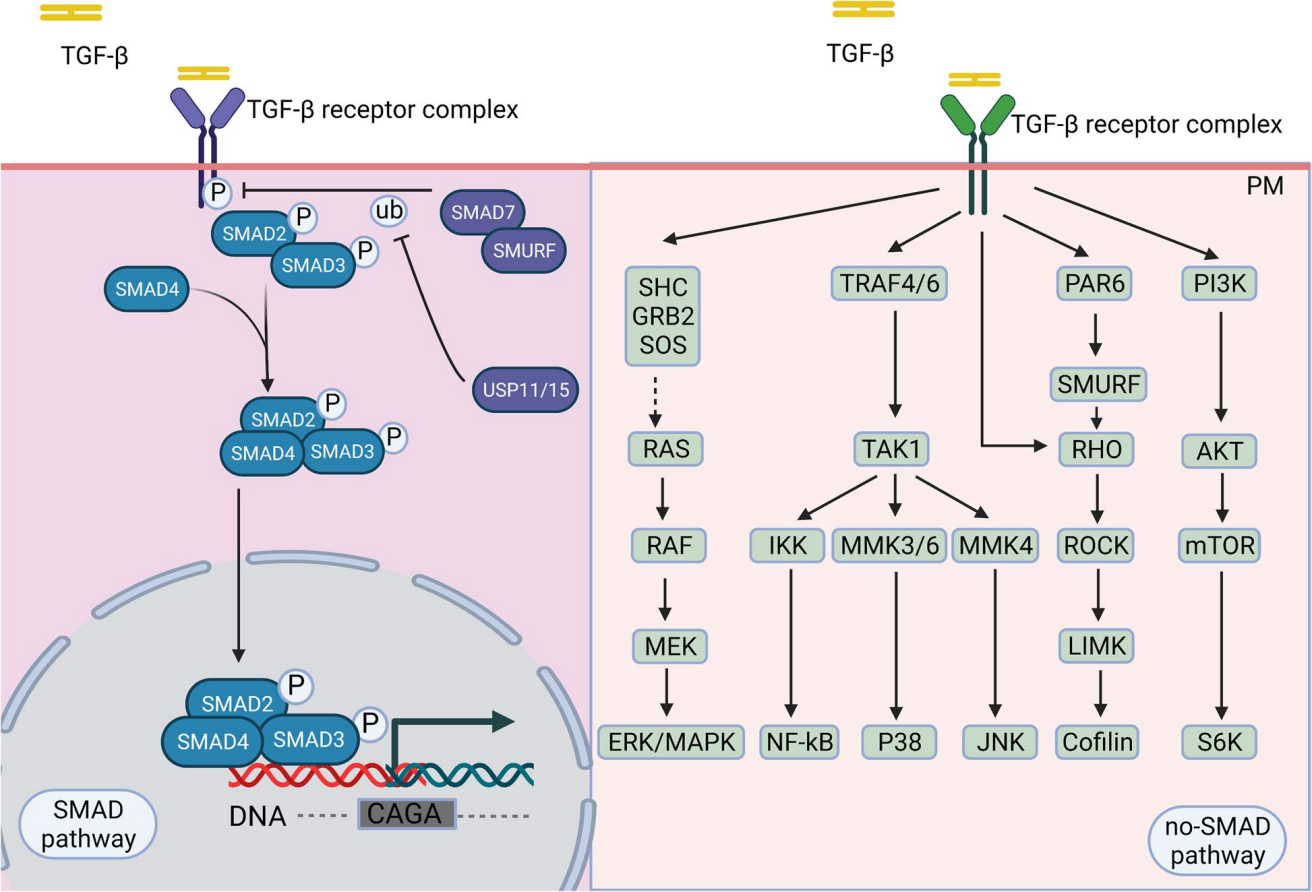
SMAD and non-SMAD pathways Schematic of the TGF-β-induced canonical SMAD and noncanonical non-SMAD signaling pathways Mature TGF-β phosphorylates TβR II, which recruits TβR I to phosphorylate receptor-SMAD proteins. Then, co-SMAD with the R-SMAD complex translates into the nucleus to regulate CAGA gene transcription. TGF-β actives non-SMAD pathways when connected to other downstream factors, such as SHC/GRB2/SODS, TRAF4/6, PAR6, and PI3K

The low-affinity heteromeric receptor complex (tβR I with tβR II) conducted by activated TGF-β stimulates different downstream signaling pathways (SMAD pathways and no-SMAD pathways) to regulate context-dependent transcription (Fig. 2). Under different physiological and pathological conditions, different kinases or signaling pathways adjust the SMAD pathway to regulate protein expression [48].

#### The canonical pathway—SMAD pathway

SMAD is a canonical pathway in which TGF-β is identified by TβR II equipped with an intracellular kinase domain, which recruits and phosphorylates TβR I with a conversed Gly/Ser-rich "GS sequence" from serine/threonine kinases. TβR II and TβR I then become a heteromeric complex [49]. Activated TβR I phosphorylates receptor-SMAD (R-SMAD) protein and promotes R-SMAD complex binding to Co-SMAD/SMAD4, forming a trimeric complex. The trimeric complex is then translated into and aggregates in the nucleus as a transcription factor to regulate target gene expression from embryonic development to adult organisms [48, 50].

In addition to being regulated by other signaling pathways or cytokines, TGF-β signaling is also automated. Downstream factors of SMAD signaling, especially Smad2/Smad3, are considered crucial mediators of TGF-β signaling in tissue fibrosis and tumorigenesis. At the same time, Smad6 and Smad7 are regarded as negative regulators to improve TGF-β-mediated fibrosis and tumorigenesis. For example, SMAD3-induced the upregulation of TSP-4, which stimulates tumor growth by mediating TGF-β-induced angiogenesis [51].

#### Noncanonical pathway—non-SMAD pathway

**Fig. 3:**
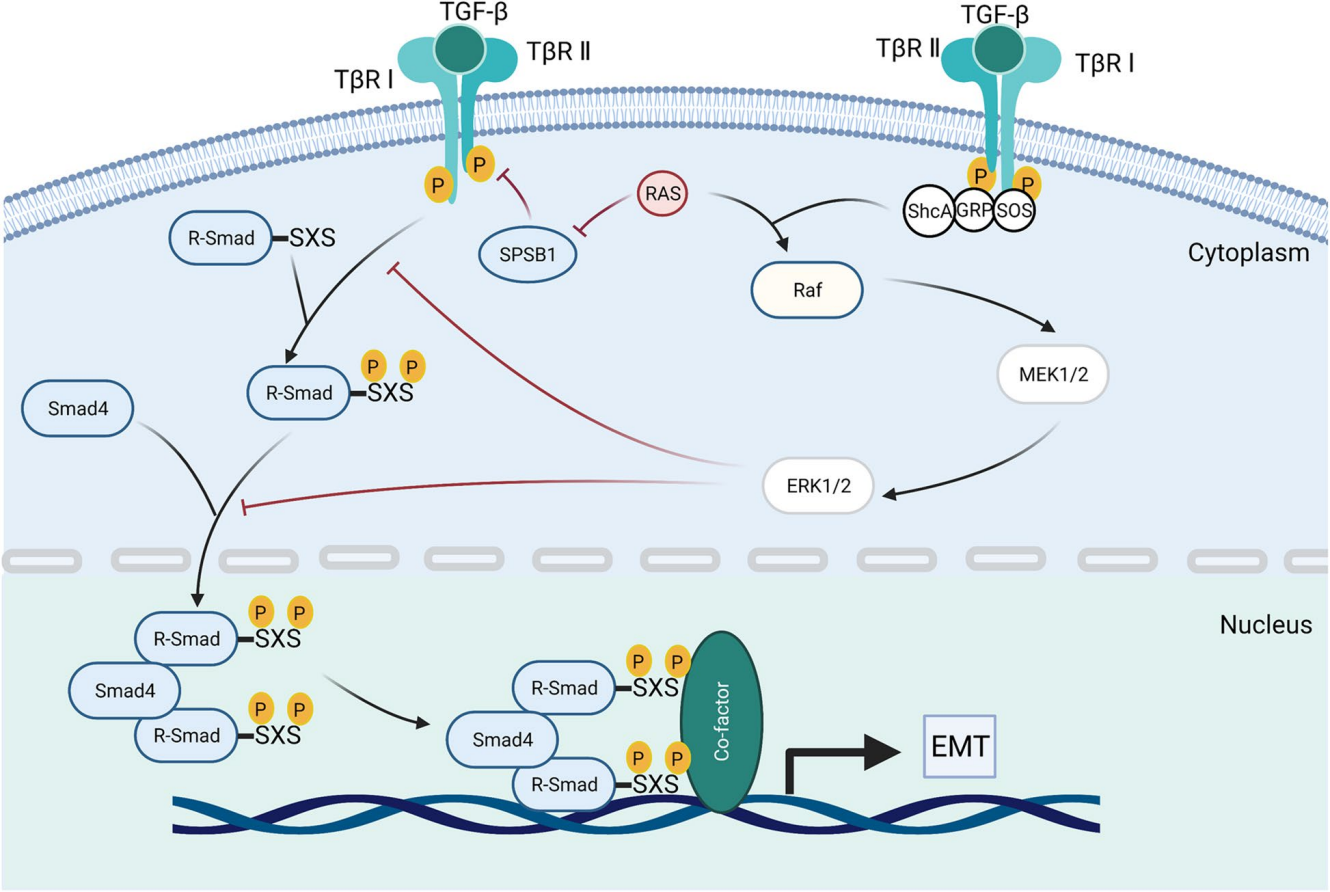
TGF-β activated the Erk MAPK pathway. Activated TβR I recruits and phosphorylates the Shc adaptor protein ShcA. Actived TGF-β promotes the formation of the ShcA/Grb2/SOS complex, Ras connects, and degrades SPSB1 via mono- and deubiquitination. TGF-β-induced GTP loading on Ras helps recruit Raf to the plasma membrane, resulting in the activation of Erk1/2 through MEKs. The activated Erk MAPK signaling pathway further influences the SMAD signaling pathway

All the pathways and downstream cascades activated by TGF-β through phosphorylation, acetylation, sumoylation, ubiquitination, and protein–protein interactions are collectively referred to as non-SMAD signaling pathways [53, 54]. These interactions mediate the intracellular responses of TGF-β and/or its related factors are collectively referred as non-SMAD signaling pathways [52, 53]. Mature TGF-β activates the mitogen-activated protein kinase (MAPK) pathway [54], extracellular signal-regulated kinases 1/2 (Erk1/2) pathways, Rho-like signaling pathways, phosphatidylinositol-3-kinase (PI3K)/AKT pathways, c-Jun amino-terminal kinase (JNK), and p38 mitogen-activated protein kinase (p38/MAPK) signaling pathways [55]. The Erk signaling pathway (Fig. 3) is essential for embryonic development in adult organisms. For instance, it affects the development of embryos, especially nerves, and EMT to promote fibrosis and cancer metastasis in geriatric diseases [56–59]. Accumulating evidence has shown that diverse TGF-β signaling responses are related to the combinatorial usage of core pathway components, including ligands, receptors, SMADs, and transcription factors by cross interacting with other pathways to regulate target gene transcription [52].

### TGF-β in fibrosis

Fibrosis is a pathological process in which organ parenchyma cell necrosis and ECM deposit excessively, causing connective tissue hyperplasia, fibrosis, or even significantly producing organ sclerosis. In addition, fibrosis is usually accompanied by the transformation of fibroblasts into myofibroblasts, even CAFs. Normal fibroblasts are components of the paraneoplastic stroma, which are critical in supporting the homeostasis of tissue-resident cells and define the architecture of organs. Several cytokines and chemokines (miR-214 [60], IL-1 [61], α-SMA, integrin β-1), and signaling pathways (EGFR, Wnt/β-catenin, Hippo, TGF-β, and JAK/STAT cascades) reprogram normal fibroblasts into CAFs [62, 63]. However, the mechanisms underlying the transformation of CAFs are rarely known.

**Fig. 4:**
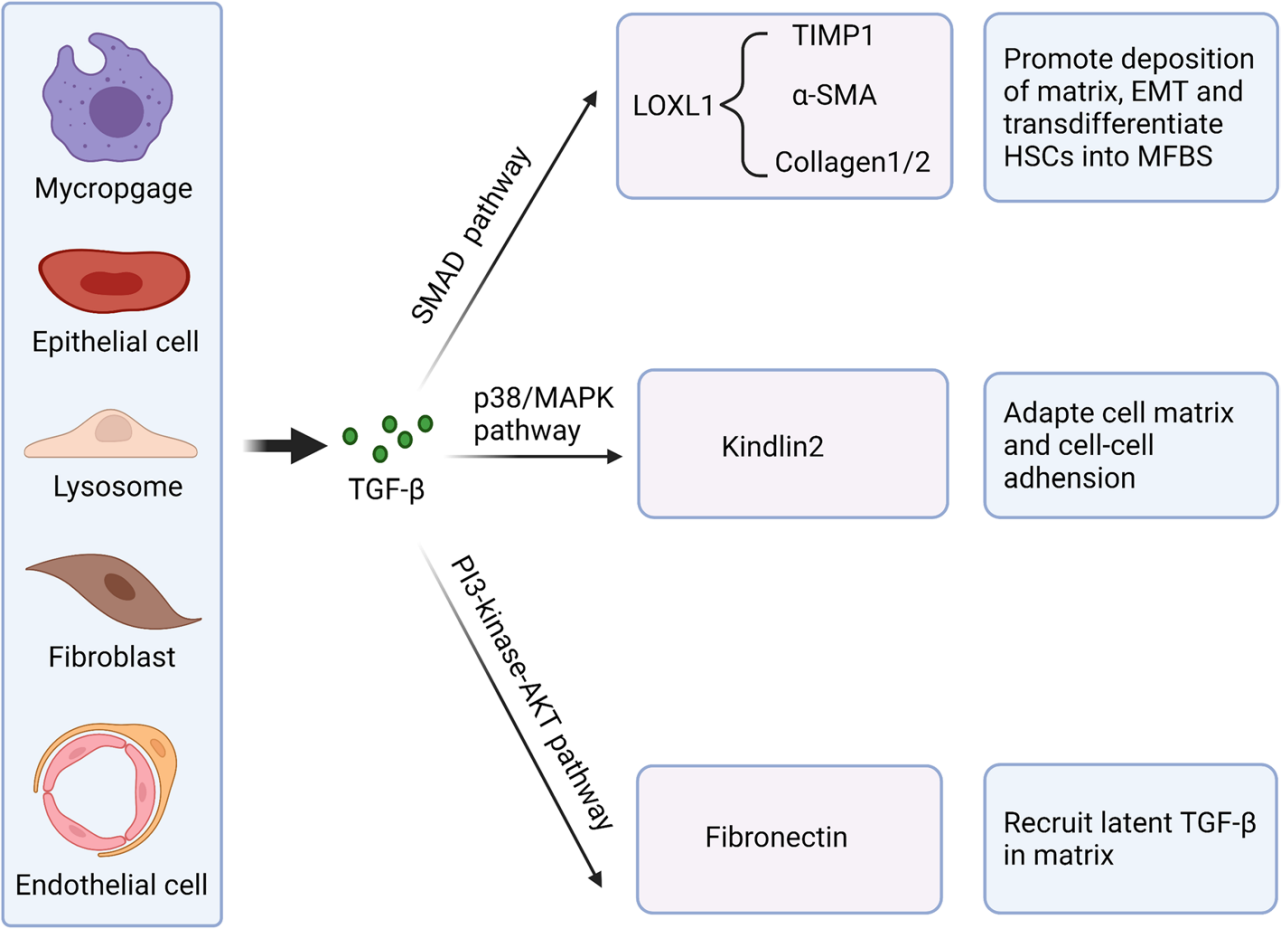
Essential functions of TGF-β in fibrosis Under pathological conditions, many different cell types, including macrophages, epithelial cells, lymphocytes, fibroblasts, and endothelial cells, can produce and secrete more TGF-β to mediate fibroblasts through SMAD and non-SMAD pathways. Although TGF-β plays a vital role in promoting fibrosis, few antifibrosis therapies target it in clinical practice

TGF-β I- III all have fibrogenic effects and share 70–82% homology at the amino acid level [64]. TGF-β I is considered as the primary factor in liver, kidney, and lung fibrosis through canonical and noncanonical signaling pathways. Usually, the cytokine TGF-β is up-regulated in tissue injury, inflammation, and wound healing [65]. The longer-term contractile state of the wound helps accelerate the expression of ECM proteins. Dysregulated TGF-β signaling promotes pathological fibrosis and tumorigenesis by excessive ECM deposition (Fig. 4). The abnormal accumulation of ECM triggers the process of fibrosis and immunosuppression by linking SMAD4, BRAF, and TP53 mutations and MYC amplification [6] and contributes to the cancer-associated fibroblast (CAF) phenotype. Scientists found that inhibiting TGF-β signaling and its downstream signaling pathways could significantly reduce fibrosis [66–68].

### TGF-β in hepatic fibrosis

Acute and chronic liver injuries promote excessive expression of TGF-β from various cell types and activation of TGF-β in the ECM. Then, activated TGF-β promotes collagenase deposition and EMT to induce fibroblast mesenchymal transformation and the activation of HSCs. In addition, increased TGF-β can be directly generated in liver injury [69]. Hepatic stellate cells (HSCs) are turned into hepatocellular carcinoma (HCC) cells [70, 71]. The activated HSCs express α-SMA but do not have lipid droplets. In addition, they give rise to myofibroblasts (MFBs), which represent the primary producer of collagen and other ECM proteins [71, 72]. 

The composition of the hepatic ECM changes during liver fibrosis and interacts with factors in TGF-β signaling to regulate hepatic fibrosis. For instance, the disruption of SMAD2 and the composition of SMAD3 promote the transcription of type II collagen toward type I and III collagen [72–74]. Meanwhile, the phosphorylation of Smad2/3 also encourages the acceleration of MMP1, α-SMA, and collagen type I, which results in the overexpression of lysyl oxidase-like 1 (LOXL1) to promote liver fibrosis [75]. Despite SMAD pathways, TGF-β also promotes HSC activation through non-SMAD pathways (MAPK, ERK, p38, and JNK pathways). For instance, activated TGF-β increases the expression of kindlin-2 via p38 and MAPK signaling, and overexpressed kindlin-2 positively feedbacks the TGF-β pathway by up-regulating Smad2 and Smad3 phosphorylation [76, 77].

Given the vital role of TGF-β in liver fibrosis, baseline TGF-β is always regarded as a biomarker of prognostic indicators. Nevertheless, clinical trials targeting TGF-β for HCC have been rare in recent years. It may be because dysregulated TGF-β cascades are not the dominate factors for HCC occurrence [78]. Galunisertib, a small-molecule selective inhibitor of TβR I, has been shown to prolong overall survival when administered with sorafenib [79]. However, it is worth noting that not all combination drug therapies help to improve HCC (NCT00557856).

### TGF-β in kidney fibrosis

Robust evidence suggests that TGF-β is a well-established central mediator of renal fibrosis. TGF-β can promote the accumulation of ECM proteins in progressive chronic kidney disease (CKD) [8, 80]. Similar to hepatic fibrosis, the development of renal fibrosis is also completed with phenotypic plasticity processes and migration, as well as invasion of epithelial cells [81], in which TGF-β has a central role. TGF-β causes progressive forms of human kidney disease by regulating apoptosis, activating ECM synthesis, and inhibiting ECM degradation through metalloproteinase inhibition [80]. TGF-β can also activate fibroblasts and translate other cell types into fibroblast-type cells directly or indirectly by SMAD or non-SMAD pathways [82, 83]. In addition, TGF-β can directly act on mesangial cells and fibroblasts to regulate cell proliferation, migration, and activation. TGF-β also mediates the accumulation of profibrotic molecules in ECM. Profibrotic molecules contain collagens, fibronectin, and plasminogen activator inhibitor-1 (PAI-1) [40, 84]. In contrast, overexpressed TGF-β indirectly prevents fibrosis. A recent study by Su J showed that TGF-β-stimulated human tubular epithelial cells and fibrotic kidneys express TGF-β/Smad3-interacting long noncoding RNA (lnc-TSI) to antagonize renal fibrosis [5].

Multiple drugs, including monoclonal antibodies (FG-3019, FG-4019), siRNAs (RXI-109, OLX-101, OLX-201), peptides (BLR-100/BLR-200), and antisense oligonucleotides, are under clinical trials, and other preclinical studies are trying to investigate more effective targets and therapies [85–87]. Furthermore, hepatocyte growth factor (HGF), BMP-7, SMAD7, and lnc-TSI can also be treated as antifibrotic targets. To date, scientists have identified multiple therapeutic targets for TGF-β-induced renal fibrosis, including microRNAs, proteins, genes, and transcription factors. For example, disrupting the recombination signal binding protein-Jκ (RBP-Jκ) could block Notch signaling, which regulates bone marrow-derived macrophages (BMDMs) to attenuate TGF-β-induced renal fibrosis [88]. MicroRNAs (miRNAs) containing 21–24 nucleotides (miR-34a, miR-30c, miR21, miR29, miR-101a, miR-34a, etc.) have been proved to play essential roles in the regulation of renal fibrosis through TGF-β signaling [89, 90]. Zhao et al. found that miR-30c inhibited the Snail 1-TGF-β axis in tubular epithelial cells to attenuate EMT, which was similar to paricalcitol [89].

### TGF-β in lung fibrosis

Idiopathic pulmonary fibrosis (IPF) is a chronic and fibrotic lung disease with a periphery to center progression, characteristic imaging, irreversible structural alterations, and tissue stiffening [91]. The observation that alveolar epithelial cells (AECs) and fibroblasts in IPF produce aberrant ECM is implicated in the TGF-β signaling pathway [92]. TGF-β is mainly derived from alveolar macrophages and metaplastic type II AECs and driven by sustained elevated mechanical tension in IPF [93]. Scientists identified the up-regulated mature TGF-β and SMAD3, SMAD4, CTGF, together with the deregulated SMAD7 in IPF [92]. Through a study of fibrotic development and glutamate metabolism, scientists found that the connection between epigenetic and transcriptional processes was almost in a TGF-β-dependent manner [94]. Despite α-SMA, TGF-β-induced integrins, MMPs, protease inhibitors, tumor necrosis factor-α (TNF-α), and regulators of small GTPases are also participated in cell-ECM interactions [95, 96]. Meanwhile, TGF-β can not only inhibit the production of antifibrotic molecules [97] but also induce serum KL6/mucin 1 (MUC1) activation [98].

TGF-β is a key profibrotic factor in IPF, but inhibiting TGF-β causes multiple side effects due to its pleiotropic effects. Though not reported in clinical trials, some TβRI kinase inhibitors showed cardiac toxicity and skin toxicity when administrated at high dose [99]. Thus, searching downstream effectors of TGF-β signaling appears to be a new research direction. Long noncoding RNAs such as RNA H19X, dynamin three opposite strand (DNM3OS), and miRNAs including 199a-5p, miR-199-3p, and miR-214-3p are all crucial to TGF-β-mediated lung fibrosis [100–103]. DNM3OS is a fibroblast-specific critical downstream effector of TGF-β-induced lung fibrosis, and interfering with it may present new effective therapeutic targets [101]. In addition, TGF-β interacts with periostin to promote lung fibrosis through the αVβ3/β5-Smad3 pathway, which can be attenuated by the integrin low-molecular-weight inhibitor CP4715 [104].

### TGF-β in cancer

TGF-β has been shown to play a crucial role in developing cancer by TGF-β pathway knockout in mice. Several experiments have demonstrated that TGF-β plays a dual role (a tumor suppressor in premalignant cells and a tumor promoter in carcinoma cells) in the process of cancer by modulating the cellular context and other effects of the cytokine [2]. TGF-β acts as a tumor suppressor by inhibiting proliferation and inducing apoptosis during the early stages of tumorigenesis [105]. Generally, TGF-β inhibits proliferation and promotes apoptosis through overexpressed cyclin-dependent kinase (CDK) inhibitors [106] and downregulated MYC expression [107]. Under this condition, premalignant cells become disseminated cancer cells, can self-impose a slow-cycling state to remain latent for extended periods [108]. The specific mechanism of how TGF-β promotes the immune escape of carcinoma cells will be described below.

Tumor cells escape antitumor surveillance of TGF-β by accumulating mutations in the TGF-β signaling cascades [109]. Examples of such escape include the mutation of SMAD4 in pancreatic ductal adenocarcinoma (PDAC) and gastric cancer (GC) [110, 111], the TβR I mutation in colon cancer [112], and even mutations in genes that encode TGF-β ligands (BMP5), receptors (TβR II, AVCR2A, BMPR2), and SMADs (SMAD2 and SMAD4) [113, 114]. Mutations in the TGF-β pathway in the head and neck, bladder, and endometrial adenocarcinomas occur in 10% to 20% of cases, compared to 25% to 50% of subjects in gastrointestinal cancer (esophageal, CRC and PDA) [111, 112, 115, 116]. Although a loss of TGF-β function mutation components is insufficient for tumor initiation, it promotes the transition of premalignant cells to a more overly malignant phenotype [2, 117].

In addition to the accumulated mutations of TGF-β signaling cascades, TGF-β-regulated immunosuppressive microenvironment also promotes tumor escape indirectly [118]. Adaptive immunity is one of three critical immune pathways implicated in disease, which is also regulated by TGF-β signaling [105, 114]. TGF-β signaling can not only control adaptive immunity by promoting the expansion of Treg cells directly, regulating the regulatory CD4 + T cell response, but also by controlling the function of effector T cells. In addition, TGF-β similarly controls the development and functions of the innate immune system by inhibiting natural killer (NK) cells [119] and regulating the proliferation of macrophages, antigen-presenting dendritic cells (DCs), and granulocytes [120]. Mutations of SMAD4 promote dysregulation of NK cell homeostasis and augment tumor cell metastases [121]. Actions on both adaptive immunity and innate immunity form a network of negative immune regulatory inputs. Luckily, scientists have indicated that TGF-β-induced immune tolerance and inflammatory responses can be flexibly treated by ionizing radiation combined with hyperthermia and checkpoint inhibitor therapies [122].

### TGF-β in melanoma

Melanoma is the most aggressive type of skin cancer, accounting for 7% of all diagnosed cancers in men and 4% in women, with approximately 7,230 fatalities in 2019 [123]. Like other cancers, as a tumor suppressor, TGF-β exerts an anti-proliferative powerful impact in normal melanocytes. As a tumor promoter, TGF-β promotes EMT, proliferation, metastasis, and immune tolerance [124, 125]. The opposite effects of TGF-β in melanoma is associated with the deregulation of cytokines (TNF-α, VEPH1, SMAD4, INF-γ, SKI) and signaling pathways (Notch1, IL-6, and Erk/MAPK pathway), which in return regulate TGF-β signaling [121, 126–133].

Adipocyte-created IL-6 and TNF-αmiR-211 promote the miR-211-repressed translation of TβR I mRNA to enhance the cellular responsiveness and metastasis of melanoma [129]. The poorly expressed ventricular zone expressed PH domain-containing 1 (VEPH1) and up-regulated upstream transcription factor 1 (USF1) in melanoma tissues promoted EMT [127, 130]. TGF-β-induced transcription sustains actomyosin force is independent of EMT [134]. TGF-β-associated VEPH1 induces proliferation, migration, and invasion of conditioned medium (CM) cells by up-regulating the expression of E-cadherin and down-regulating the expression of N-cadherin, Vimentin, and SMAD4 [130, 135]. Notably, SMAD4 suppresses tumor metastasis and promotes antitumor immunity through up-regulated IFN-γ and granzyme B (GZMB) by non-SMAD in NK cells at early stages [119, 121]. Immune cells, such as TGF-β-sustained effector T cells, secrete CD73 to facilitate tumor resistance of anti-CD137 therapy [136]. In BRAF (V600E)-mutant melanoma, the sex-determining region Y-box 10 (SOX10) is suppressed, and BRAF signaling-activated TFEB S142 phosphorylation is promoted. Both of them help increase melanoma metastatic potential and drug resistance [137, 138].

Therapies targeting these deregulated cytokines and signaling pathways combined with radiation, chemotherapy, and other targeted therapies become revolutionary therapeutic strategies. In addition to MECOM and BMP5 in BRAF-mutated melanoma, GNAQ or CNA11 mutations in uveal melanoma are also associated with TGF-β signaling [139]. Furthermore, GNAQ or CNA11 mutations demonstrate low sensitivity or resistance to specific treatments [140, 141]. They indicate a suite of rationally designed clinical trials and potentially clinical targets. Scientists indicated that hydrophobic TGF-β inhibitor (SB-505124) and an adenoviral vector expressing IL-12 increase animal survival [142]. PD-1/PD-L1 antigen-specific checkpoints block siRNA entry into antigen-presenting cells. In addition, PD-1/PD-L1 antigen-specific checkpoints are associated with lipid-coated calcium phosphate (LCP) mRNA vaccine, which indicates a more robust immune response to melanoma growth and metastasis [143]. Overall, the rational development of multiple anticancer therapies, such as the combination of TGF-β inhibitors with checkpoint inhibitors and/or other biological treatments, holds excellent prospects.

### TGF-β in pancreatic ductal adenocarcinoma

Pancreatic ductal adenocarcinoma (PDAC) is the most aggressive type of gastrointestinal tumor due to its rapid progression and resistance to traditional chemoradiotherapy [144]. Studies have shown that whether TGF-β acts as a tumor suppressor or a tumor promoter depends on the tumor microenvironment [145]. In the early stage of pancreatic cancer, TGF-β promotes apoptosis via ID1 [146], regulates cell cycle progression through G1 arrest [147], and inhibits the growth of epithelial cells. In addition, a decrease in VEGF and an increase in TSP-1 caused by TGF-β help inhibit pancreatic cancer [148]. However, during the advanced stage of PDAC, genetically inactivated TGF-β signaling has a potent growth promotor effect [149, 150]. Of note, TGF-β does not only promote evasion and metastasis in all advanced pancreatic cancer. Overexpressed TGF-β drives tumor suppression in SMAD4-positive PDA cells by repressing KLF5 [151].

More evidence is emerging that at least one mutation in the TGF-β signaling genes (TGFβRI, TGFβRII, Smad2, and Smad4 genes) occurs in all PDAC [152, 153]. SMAD mutation occurs in 60% of pancreatic cancer patients. An increased KRAS mutation and SMAD mutation in PDAC patients drive early tumorigenesis and metastasis. [154–156]. The mutated TGF-β signaling pathway has a much stronger ability to inhibit proliferation, promote angiogenesis and immune escape than simply shutting down the TGF-β signaling pathways [157, 158]. SMAD4 deletion leads to up-regulation of the oncogene (PGK1) [159]and down-regulation of the anticancer gene (SMAD4/DPC4). Such regulation promotes cell metastasis [148]. Meanwhile, deregulated TGF-β signaling leads to ECM deposition and immunosuppressive cell infiltration [160–162]. This kind of deposition and infiltration accelerates the metastasis of pancreatic cancer cells and rationalizes early PDAC dissemination [163, 164].

TGF-β plays a crucial role in the process and metastasis of PDAC, and therapies targeting TGF-β signaling hold great promise. Several strategies relevant to TGF-β signaling have been investigated in preclinical and clinical researches and have shown efficacy partially [165–167]. Therapeutic approaches are always associated with three levels of ligand, ligand-receptor binding, and intracellular transduction to disrupt TGF-β signaling. These approaches contain TβR II antagonists, sequence-targeted antifibrosis nanoparticles, anti-TGF-β recombinant protein, and DC vaccines [168–170]. Lipoxin A4 (LXA4), a metabolite derived from arachidonic acid, could significantly inhibit TGF-β signaling in PDAC [171]. Strategies targeting ligand-receptor binding levels, such as TGF-β inhibitors and monoclonal blocking antibodies, also show robust performance against PDAC [172]. TGF-β inhibitors are primarily TβR-targeted and SMADs-associated kinases at signal cell level. The most effective treatment is the combination of TGF-β inhibitors with chemotherapy and other biological agents. For example, vactosertib (activin receptor-like kinase 5 inhibitor) [173] in combination with nal-IRI plus 5-Fluorouracil/Leucovorin improved overall survival rates compared with vactosertib alone [174, 175]. Nanotargeted relaxin, an endogenous hormone, has also been shown to enhance the efficacy of gemcitabine in vivo [176]. Furthermore, the selection of correct dosage form and the establishment of a demonstration drug delivery system are critical for the treatment of desmoplastic tumors. Compared with traditional Chinese medicine dosage forms (decoction and powder), the targeted administration of nano-preparations (α-mangostein and triptolide) can overcome the permeation obstacles in PDAC and improve therapeutic effects. [176].

### TGF-β in colorectal cancer

Colorectal cancer (CRC) is the leading cause of death among cancers of the digestive system (101,420 estimated new cases and 51,020 estimated deaths in 2019) [123], the poor prognosis of which is mainly associated with colorectal cancer metastasis and immune evasion. Many studies have indicated that malignant CRC is characterized by high stromal infiltration with innate immune cells, fibroblasts, and TGF-β activation [177]. TGF-β is involved in regulating CRC metastasis, tumor stroma, microenvironment, and immune system resistance.

Colorectal cancer is driven by the accumulation of mutations in APC, KRAS, TβR II, Trp53 [178–182], carcinoembryonic antigen-associated cell adhesion molecules (CEACAM) [183] and R-spondins (RSPOs) [184]. The four primary [185]mutations in intestinal tumors promote CRC metastasis, indicating a negative prognostic effect for recurrence of CRC [186–188] and regulating the tumor microenvironment [112, 189]. Despite these mutations demonstrate worse clinical outcomes, they also predict neoantigen-specific immunotherapeutic anti-TGF-β strategies [187].

It has been confirmed that inhibiting TGF-β signaling pathways in the preclinical and clinical treatment of CRC are effective [190]. However, anti-TGF-β therapy alone is insufficient to mediate antitumor immunity in CRC. In contrast, the combination of other biological agents or irradiated tumor vaccine with anti-TGF-β treatment can reduce CRC metastasis. Chemotherapies ginsenoside Rb2 [191] and tanshinone II A [192] showed therapeutic effects on CRC by inhibiting TGF-β-induced EMT and angiogenesis, respectively [193]. Nevertheless, the effect was mild. Monotherapy with galunisertib (LY2157299), an oral small-molecule inhibitor of the TβR I kinase, was also not significant [194]. Coadministration of TGF-β blocking agents and anti-PD-L1 antibodies indicated a dramatic response by promoting CD8 + T cells penetration into the tumor [189].

### TGF-β in breast cancer

Along with lung and colon cancer, breast cancer is one of the most common cancers worldwide and is more malignant in females than in males. Although the mortality rates of breast cancer are decreasing in some developed countries, there are approximately 500,000 deaths because of breast cancer every year [195, 196]. Further understandment of the development, progression, and treatment of breast cancer is emergency.

The TGF-β signaling pathway is well known to play a vital role in cancer regulation, and breast cancer is no exception [115, 157]. TGF-β regulates the survival of cancer cells to influence breast cancer progression. On the one hand, TGF-β can induce the anti-apoptotic effects of mouse mammary carcinoma cells through up-regulated chondrocytes 1. Chondrocytes 1 is a basic helix-loop-helix (bHLH) transcription factor, which is tightly related to breast carcinomas [197]. On the other hand, the TGF-β signaling pathway can also disturb the immune system to induce immune evasion. In breast cancer, the lack of TβR III and its shed extracellular domain (sTβR III) will enhance TGF-β signaling within DCs. It finally results in Tregs infiltration and immune suppression [198]. In addition, TGF-β can also transactivate EGFR through the Smad3 and ERK/Sp1 signaling pathways to promote the migration and proliferation of breast cancer cells [199].

Moreover, we should highlight the contribution of TGF-β to breast cancer metastasis because breast cancer can quickly metastasize to the lung, brain, bone, and liver, which is lethal [200]. In addition to breast cancer, TGF-β is also critical in the metastasis of other cancers including bone, and gastric cancer [201–203]. TGF-β participates in breast cancer metastasis by up-regulating CXCR4 in monocytes. These attracted and differentiated tumor-associated macrophages (TAMs) assist tumor cell extravasation [204]. Additionally, miR-190 and OTU domain-containing protein 1 are two inhibitors of TGF-β signaling that target SMAD2 and SMAD7, respectively. The expression of SMAD2 and SMAD7 is associated with outcomes in breast cancer patients, for downregulated SMAD2 and SMAD7 promote breast cancer metastasis [205, 206].

The mechanism of TGF-β promoting breast cancer is manifold. Therefore, targeting TGF-β signaling is probably an effective way to treat breast cancer. Artemisinin derivatives, like artesunate (ARS) and dihydroartemisinin (DHA), are effective in suppressing TGF-β signaling and CAF activation. Breast cancer will be in remission because of the reduced interaction between the tumor and tumor microenvironment [207]. In addition, a bispecific receptor decoy containing TGF-β neutralizing the TβR II extracellular domain was designed. This decoy and ibalizumab were intended recently to inhibit TGF-β signaling in TH cells and decrease tumor burden in a breast cancer mouse model [208]. Due to the deficiency of SIRT7 in breast cancer metastasis mice, TGF-β signaling is activated to promote metastasis. It is already clear that resveratrol can activate SIRT7, regulate SMAD4 deacetylation, and most importantly inhibit metastasis [209].

### TGF-β in glioma cancer

Glioma is a malignant primary brain tumor divided into four categories, including circumscribed gliomas (WHO grade I) and diffusely infiltrating gliomas (WHO grade II-IV). Diffusely infiltrating gliomas are more malignant than circumscribed gliomas, in which glioblastoma is the most lethal glioma, with a median overall survival of 14–17 months [210, 211].

Among the numerous signaling pathways that play a role in glioma, TGF-β signaling is being noted. The related mechanism and therapeutic strategies have been gradually clarified. It has already been found that the high proliferation and invasion of gliomas and the poor prognosis in glioma patients are usually accompanied by SMAD signaling in early studies, and Sox9 becomes an important regulatory target when TGF-β works in glioma progression [212, 213]. TGF-β plays an essential role in glioma progression by inducing the proliferation, invasion, EMT, and migration of glioma cells and depressing immune effector cells [214–216]. Furthermore, three kinds of TGF-β are all related to glioma. In a study of the relationship between TAMs and the progression of tumors, Z. Liu et al. found M2 phenotype TAMs to promote the stemness and migration of glioma cells by secreting TGF-β [217]. In addition, TGF-βII affects autophagy, a vital process connected with tumor growth, promoting glioma cells' invasion through the SMAD and non-SMAD pathways [218, 219]. Among those three isoforms, the expression of TGF-βIII was lower than that of the other two isoforms. However, it has an essential effect on SMAD phosphorylation and tumor invasiveness [220].

Previous studies have shown that overexpressed TGF-β in the glioma is involved in angiogenesis, tissue invasion, and cancer progression. Therapies targeting TGF-β are divided into three levels: TGF-β mRNA translation inhibitors, TGF-β neutralizing antibodies and receptor inhibitors, and regulators of TGF-β signaling pathway downstream factors. In a phase II clinical study (NCT00431561), intratumorally administered AP12009 alone exhibits one-third of the efficacy population [221]. AP12009 is a phosphorothioate antisense oligodeoxynucleotide specific for the mRNA of human TGF-βII [221]. RGFP966, along with an HDAC3 inhibitor, regulated SMAD7 acetylation rather than ubiquitination to promote gastric stump carcinoma (GSC) differentiation [222]. There appeared to be no difference in efficacy between monotherapy of TGF-β antibodies (GC1008, NCT01472731) or small-molecule TβR I inhibitors (LY2157299, NCT01220271), and their combination with chemotherapy (Table 1) [223]. The exploration of appropriate combination therapy is still the mainstream direction.

**Table 1:** Therapies targeting TGF-β signaling under clinical trials in the past 3 years

Drug	Mechanism	Indication	Development stage	ClinicalTrials.gov identifier
SAR439459	Pan- TGFβ neutralizing antibody	Advanced Malignant Solid Neoplasm/Metastatic Malignant Solid Neoplasm/Unresectable Malignant Solid Neoplasm	Phase 1	NCT04729725
		Plasma Cell Myeloma Refractory	Phase1/Phase 2	NCT04643002
		Advanced Liver Cancers	Phase1/Phase 2	NCT04524871
NIS793	Fully human anti-TGF-β IgG2 monoclonal antibody	Metastatic Pancreatic Ductal Adenocarcinoma	Phase2/Phase 3	NCT04390763/NCT04935359
		Myelofibrosis	Phase1/Phase 2	NCT04097821
		Myelodysplastic Syndromes	Phase 1	NCT04810611
ABBV151	Humanized monoclonal antibody inhibitor of GARP- TGF-β1	Advanced Solid Tumors Cancer	Phase 1	NCT03821935
AVID200	Engineered TGF-β ligand trap	Malignant Solid Tumor	Phase 1	NCT03834662
		Primary Myelofibrosis/Post-essential Thrombocythemia Myelofibrosis/Post-polycythemia Vera Myelofibrosis	Phase 1	NCT03895112
		Scleroderma, Diffuse	Phase 1	NCT03831438
M7824 (bintrafusp alfa)	Bifunctional anti-PD-L1/TGF-βRII Trap fusion protein	Thymic Epithelial Tumor/Recurrent Thymoma/Thymic Cancer	Phase 2	NCT04417660
		Metastatic Colorectal Cancer/Advanced Solid Tumors With Microsatellite Instability	Phase1/Phase 2	NCT03436563
		HPV Positive Cancer	Phase1/Phase 2	NCT04432597
		Urothelial Cancer	Phase 2	NCT04501094
		Kaposi Sarcoma	Phase1/Phase 2	NCT04303117
		Urothelial Cancer/Bladder Cancer/Genitourinary Cancer/Urogenital Neoplasms/Urogenital Cancer	Phase 1	NCT04235777
		Advanced Pancreas Cancer	Phase1/Phase 2	NCT04327986
		Mesothelioma; Lung	Phase 2	NCT05005429
		Stage II-III HER2 Positive Breast Cancer	Phase 1	NCT03620201
		Relapsed Small Cell Lung Cancers	Phase1/Phase 2	NCT03554473
		Unresectable Stage III Non-Small-Cell Lung Cancer	Phase 2	NCT03840902
		Advanced Stage Breast Cancer	Phase 1	NCT04296942
		Prostate Neoplasms	Phase1/Phase 2	NCT04633252
		Metastatic Triple-Negative Breast Cancer	Phase 1	NCT03579472
		Advanced Solid Tumors	Phase1/Phase 2	NCT04574583
		Metastatic Prostate Cancer/Advanced Solid Tumors	Phase1/Phase 2	NCT03493945
		Advanced HPV Associated Malignancies	Phase1/Phase 2	NCT04287868
		Metastatic Checkpoint Refractory HPV Associated Malignancies/Microsatellite Stable Colon Cancer (MSS)	Phase 1/Phase 2	NCT04708470
		Triple-Negative Breast Neoplasms	Phase 2	NCT04489940
		Small Bowel Cancers/Colorectal Cancers	Phase 2	NCT04491955
		Esophageal Squamous Cell Carcinoma	Phase 2	NCT04595149
		Untreated Resectable Non-Small-Cell Lung Cancer	Phase 2	NCT04560686
		Cancers With Brain Metastases	Phase1/Phase 2	NCT04789668
		Recurrent Head and Neck Squamous Cell Carcinoma/Second Primary Squamous Cell Carcinoma of the Head and Neck	Phase1/Phase 2	NCT04220775
		Metastatic or Locally Advanced Urothelial Cancer	Phase 1	NCT04349280
		Squamous Cell Carcinoma of Head and Neck	Phase 2	NCT04428047
		Biliary Tract Cancer/Cholangiocarcinoma	Phase 2	NCT04727541
		Advanced Non-small-Cell Lung Cancer	Phase 2	NCT04396535
		Locally Advanced or Metastatic Tyrosine Kinase Inhibitor-Resistant EGFR-Mutant Non-small-Cell Lung Cancer	Phase 2	NCT04971187
GFH018	Inhibitor of TGF-βRI	Advanced Solid Tumor	Phase1/Phase 2	NCT04914286
SHR-1701	Bifunctional anti-PD-L1/TGF-βRII agent	Pancreatic Cancer	Phase1/Phase 2	NCT04624217
		Metastatic or Locally Advanced Solid Tumors	Phase 1	NCT03710265/NCT03774979
		Advanced Solid Tumors	Phase1/Phase 2	NCT04856774
		Nasopharyngeal Carcinoma	Phase 1	NCT04282070
		Advanced Solid Tumors	Phase 1	NCT04324814
		Metastatic Colorectal Cancer	Phase2/Phase 3	NCT04856787
		Advanced Solid Tumors and B-cell Lymphomas	Phase1/Phase 2	NCT04407741
JS201	Recombinant PD-1 monoclonal antibody/TGF-βRII bifunctional fusion protein	Advanced Malignant Tumors	Phase 1	NCT04956926
		Small-cell Lung Cancer	Phase 2	NCT04951947
TST005	Bispecific antibody consisting of a PD-L1 monoclonal antibody (mAb) and a TGF-β trap	Locally Advanced or Metastatic Cancers/Metastatic Human Papillomavirus-Related Malignant Neoplasm	Phase 1	NCT04958434
TASO-001	Antisense oligonucleotide against TGF-β2	Advanced or Metastatic Solid Tumor	Phase 1	NCT04862767
TEW-7197 (Vactosertib)	TGF-β receptor ALK4/ALK5 inhibitor	Metastatic Pancreatic Cancer	Phase1/Phase 2	NCT03666832
		Advanced Stage Solid Tumors	Phase 1	NCT02160106
		Myeloproliferative Neoplasm	Phase 2	NCT04103645
LY2157299 (galunisertib)	Small molecule antagonist of the tyrosine kinase TGFBR1	Nasopharyngeal Carcinoma	Phase 2	NCT04605562
LY3200882	Inhibitor of TGFβRI	Solid Tumor	Phase 1	NCT02937272
TRK250	siRNA-based oligonucleotide selectively suppressing TGFβ1	Idiopathic Pulmonary Fibrosis	Phase 1	NCT03727802
STP705	siRNA-based oligonucleotide selectively suppressing TGFβ1 and COX-2	Basal Cell Carcinoma	Phase 2	NCT04669808
		Bowen's Disease/Cutaneous Squamous Cell Carcinoma in Situ	Phase1/Phase 2	NCT04293679
		Keloid	Phase 2	NCT04844840
		Hepatocellular Carcinoma/Liver Metastases/Cholangiocarcinoma	Phase 1	NCT04676633
		Squamous Cell Carcinoma in Situ	Phase 2	NCT04844983
QLS31901	PDL1/TGFβ antibody	Advanced Malignant Tumor	Phase 1	NCT04954456
ACE-1334	superfamily based ligand trap of TGFβ1 and c3	Systemic Sclerosis With and Without Interstitial Lung Disease	Phase 1/Phase 2	NCT04948554
ACE-536 (Luspatercept)	TGFβ superfamily ligand trap	Myelodysplastic Syndromes	Phase2/Phase 3	NCT04477850/NCT03900715/NCT03682536
		Myelodysplastic Syndromes/Β-thalassemia/Myeloproliferative Neoplasm-Associated Myelofibrosis	Phase 3	NCT04064060
		Myeloproliferative Disorders/Myelofibrosis/Primary Myelofibrosis/Post-Polycythemia Vera Myelofibrosis/Anemia	Phase 3	NCT04717414
		Β-Thalassemia	Phase 2	NCT04143724
		Primary Myelofibrosis/Post-Polycythemia Vera/Myelofibrosis	Phase 3	NCT03755518
NNC0361-0041	Recombinant supercoiled plasmid encoding PPI, TGF-β1, IL-10, and IL-2	Type I Diabetes	Phase 1	NCT04279613
PF-06952229	TGFβ1 inhibitor	Advanced Solid Tumors	Phase 1	NCT03685591
GT90001	Fully human anti-ALK-1 mAb (IgG2)	Metastatic Hepatocellular Carcinoma	Phase1/Phase 2	NCT03893695
		Solid Tumors	Phase1/Phase 2	NCT04984668
Trabedersen	TGFβ2 specific phosphorothioate antisense oligodeoxynucleotide	COVID-19	Phase 2	NCT04801017

### Clinical applications of TGF-β-targeting therapies

Extensive evidence suggests that targeting TGF-β cascades has the potential to treat patients with fibrosis and cancers. Numerous anti-cancer and anti-fibrosis pharmacological interventions targeting TGF-β have undergone pre-clinical and clinical stages. TGF-β-targeted drugs are mainly divided into neutralizing antibodies, small-molecule TGF-β inhibitors, ligand traps, antisense oligonucleotides, and vaccines (Table 1) [224]. Among all the TGF-β targeting drugs, Fresolimumab (GC1008), Galunisertib (LY2157299), Trabedersen (AP12009), and Vactosertib are the most striking drugs [224–226]. Moreover, Trabedersen, a TGF-βII specific phosphonothioate antisense oligodeoxynucleotide, also demonstrated a therapeutic effect on COVID-19 (NCT04801017).

Despite the encouraging potential displayed by TGF-β-targeted drugs in a part of pre-clinical animal studies, the results from subsequent clinical trials of those drugs seem to be disappointing. The application of TGF-β inhibition strategies in patients with fibrosis is challenging due to the systemic effects of TGF-β and the complexity of cancer and fibrosis formation [227]. Firstly, although TGF-β cascades are commonly activated to contribute to pathological processes, the physiological function of TGF-β cannot be ignored. Therefore, the wide defection of TGF-β may lead to the disturbance of normal physiological processes, which should be treated with caution [228]. Secondly, TGF-β modulates a wide range of signaling cascades to promote fibrosis and cancers, which increases the difficulty and complexity of the treatment. Exploring precise downstream TGF-β-activated factors for each disease is necessary. Thirdly, despite the key role of TGF-β in fibrosis and tumorigenesis, the onset and development of the disease is multifactorial. The combinational therapeutic strategies of TGF-β-targeted therapy with other traditional ones should be studied to achieve an ideal effect.

## Conclusion

TGF-β plays a vital role from early embryonic development to adult homeostasis. However, dysregulation of TGF-β signaling is significantly associated with tumorigenesis and fibrosis. The exact mechanism is complex and mainly involves TGF-β as a tumor suppressor in premalignant cells and a tumor promoter in carcinoma cells by regulating EMT, ECM accumulation, immune invasion, and CAFs activation. TGF-β overexpression under pathological conditions directly promotes tissue lesions. In addition, TGF-β signaling cascade group mutation accumulation is also closely related to fibrosis and tumorigenesis.

The twenty-first century has witnessed a significant upgrade of precision medicine, among all, targeted therapy as the most promising one. Lots of preclinical researches have demonstrated the efficacy of TGF-β related pharmacological agents. In recent years, there have been various clinical experiments evaluating TGF-β-targeted antibody, small molecular receptor inhibitors, ligand traps, antisense oligonucleotides, and vaccines. Unfortunately, anti-TGF-β approaches achieved subtle efficacy due to the systemic biological effects of TGF-β and the complexity of fibrosis and tumorigenesis. It is known that most cancer patients die of metastasis after chemotherapy or radiotherapy, where the immunosuppressive TGF-β in the TME might be one of the factors. Therefore, the combination therapy of chemotherapy/ radiotherapy/targeted therapy with TGF-β-targeted therapies might be developed to achieve an enhanced antitumor efficacy by regulating tumor microenvironment. In addition, in future researches, researchers should further focus on the optimization of dosing and drug delivery systems in TGF-β-related therapies. Above all, the exploration of comprehensive mechanisms of TGF-β in diseases and the development of TGF-β based combination therapies might be very crucial for combatting fibrosis and cancer in future.

## Data Availability

Not applicable.

## Abbreviations

TGF-β: Transforming growth factor β
BMPs: Bone morphogenetic proteins
GDFs: Growth and differentiation factors
ECM: Extracellular matrix
TβR: TGF-β receptor
EMT: Epithelial mesenchymal transition
PD-L1: Programmed cell death 1 ligand 1
LAP: Latency associated peptide
LLC: Large latent complex
LTBP: Latent TGF-β binding proteins
TSP-1: Thrombospondin 1
GARP: Glycoprotein a repetitions predominant protein
SMAD: Drosophila mothers against decapentaplegic
SLC: Small latent complex
LTBP: Latent TGF-β binding proteins
MMP-2: Matrix metalloproteinase-2
RGD: Arginine-glycine-aspartic acid
Tregs: Regulatory T cells
ASCs: Adipose-derived mesenchymal stem cell
R-SMAD: Receptor complex phosphorylates receptor-SMAD
LMO7: LIM domain only 7
IL-2: Interleukin-2
CDK: Cyclin-dependent kinase
HK2: Hexokinase2
MAPK: Mitogen-activated protein kinase
Erk: Extracellular signal regulated kinases
PI3K: Phosphatidylinositol-3-kinase
JNK: C-Jun amino terminal kinase
CAFs: Cancer-associated fibroblasts
TIMP: Tissue inhibitor of metalloproteinases
α-SMA: α-Smooth muscle actin
LOXL1: Lysyl oxidase-like 1
HSCs: Hepatic stellate cells
MFBs: Myofibroblasts
HCC: Hepatocellular carcinoma
MFBs: Myofibroblasts
CTGF: Connective tissue growth factor
KCs: Kupffer cells
CKD: Chronic kidney diseases
PAI-1: Plasminogen activator inhibitor-1
UUO: Unilateral ureteral obstruction
HGF: Hepatocyte growth factor
RBP-Jκ: Recombination signal binding protein-Jκ
miRNAs: MicroRNAs
PTHrP: Parathyroid hormone-related protein
EGF: Epidermal growth factor
VEGF: Vascular endothelial growth factor
IPF: Idiopathic pulmonary fibrosis
AECs: Alveolar epithelial cells
TNF-α: Tumor necrosis factor-α
MUC1: Mucin 1
DNM3OS: Dynamin 3 opposite strand
PDAC: Pancreatic ductal adenocarcinoma
GC: Gastric cancer
DCs: Dendritic cells
VEPH1: Ventricular zone expressed PH domain-containing 1
USF1: Upstream transcription factor 1
CM: Conditioned medium
GZMB: Granzyme B
SOX10: Sex determining region Y-box 10
LCP: Lipid-coated calcium phosphate
PDAC: Pancreatic ductal adenocarcinoma
LXA4: Lipoxin A4
CRC: Colorectal cancer
CEACAM: Carcinoembryonic antigen-related cell adhesion molecule
RSPOs: R-spondins
bHLH: Basic helix-loop-helix
sTβR III: Shed extracellular domain
TAMs: Tumor-associated macrophages
ARS: Artesunate
DHA: Dihydroartemisinin
GSC: Gastric stump carcinoma
